# Supplementary material for: More isn’t always better: Higher exoskeleton torque does not consistently improve balance performance
Source: J Biomech. Author manuscript; Available in PMC 2026 Oct 26. (PMC13702090; doi:10.1016/j.jbiomech.2026.113574)
Supplement: Supplementary file 1 [file NIHMS2216624-supplement-Supplemental.pdf]

SUPPLEMENTAL

Extended methods - Delayed CoM feedback model

To use the delayed CoM feedback model, all trials from the NO torque condition were averaged to generate the average CoM kinematics and inverse dynamics-derived ankle joint torque. The average curves were then used for all subsequent analyses. To isolate reactive ankle torque, background torque (defined as the mean ankle torque one second before platform movement) was subtracted before optimizing gains and delays. For each of the two loops, optimization using *fmincon.m* was performed to identify K_{Di}, K_{Vi}, K_{Ai}, and λ_i, where i indicates the ith loop. Parameter bounds were imposed on each loop to prevent the algorithm from searching outside a physiologically relevant space and reconstructing the same ankle torque curve features. During the fitting process, the fit of each loop was evaluated. If the loop poorly reconstructed the data, hand-tuning of the bounds was performed to achieve the best model as quantified by the coefficient of determination (R^2) and variance accounted for (*VAF*). After two separate optimizations identified the best gains for each loop, the gains and delays were concatenated as the initial guess for a final optimization. The final optimization concurrently optimized the gains for both loops. In this optimization, the lower and upper bounds were restricted to ±10% of the initial optimization, while the delays were restricted to ±10ms of the initial optimization.

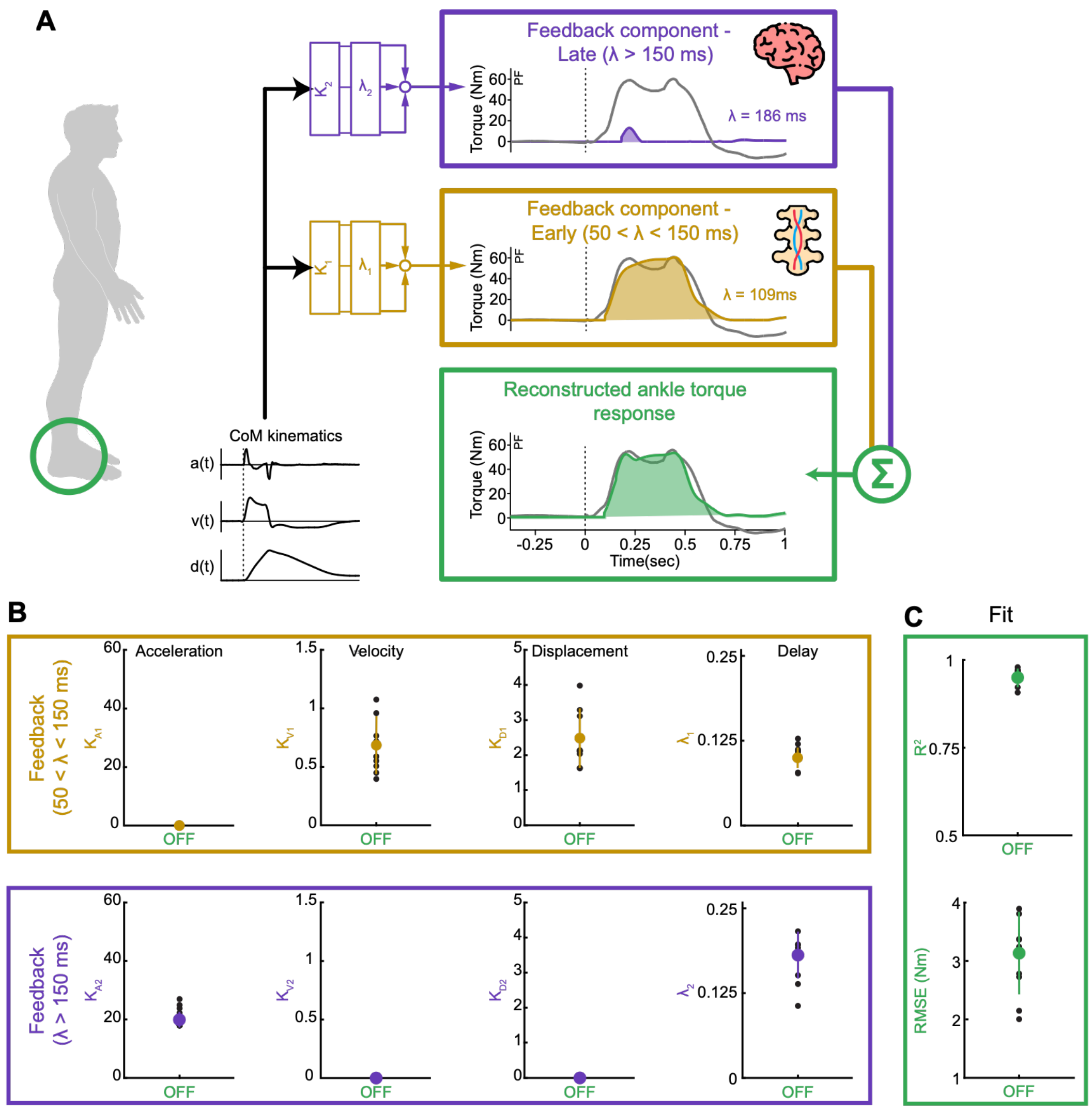

Supplementary Figure 1. (A) The delayed center of mass (CoM) feedback model for the ankle torque response, indicating that the response is mediated by an "early" (delay < 150 ms; gold) and "late" (delay > 150 ms; purple) feedback component. These two components are summed together, resulting in the overall ankle torque response (green). (B) The delayed CoM feedback model gains for each loop. K_A, K_V, and K_D represent the CoM displacement, velocity, and acceleration gains, while λ is the time delay. (C) The delayed CoM feedback model reconstructed the torque response well, with a high R^2 and low *RMSE*. (B-C) The colored dots represent the mean, and the colored vertical lines represent the standard deviation of the optimized gains. The black dots represent each participant.

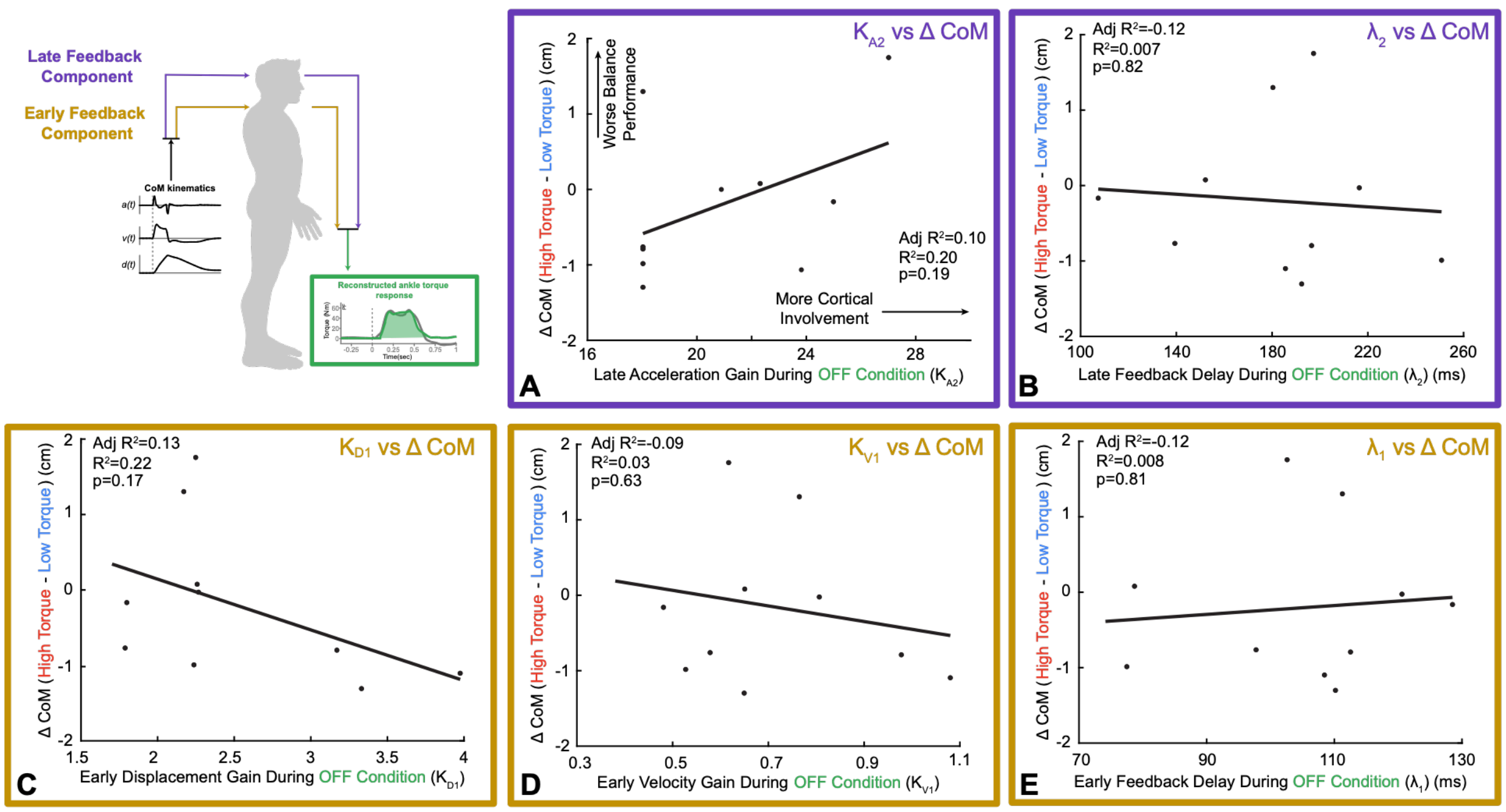

Supplementary Figure 2. (A-E) The difference in CoM displacement between the HIGH torque and LOW torque conditions (ΔCoM) was not related to any of the optimized feedback gains or delays. The solid black line indicates the linear fit. The black dots represent each participant.
